# Mental Health Care Professionals’ Appraisal of Patients’ Use of Web-Based Access to Their Electronic Health Record: Qualitative Study

**DOI:** 10.2196/28045

**Published:** 2021-08-27

**Authors:** Antonius Mattheus van Rijt, Pauline Hulter, Anne Marie Weggelaar-Jansen, Kees Ahaus, Bettine Pluut

**Affiliations:** 1 Erasmus School of Health Policy & Management Erasmus University Rotterdam Rotterdam Netherlands; 2 Eindhoven University of Technology Eindhoven Netherlands

**Keywords:** patient portals, eHealth, mental health care professionals, mental health, eMental health, mental health care, patient-accessible, electronic health records, Open Notes, normalization process theory, NPT

## Abstract

**Background:**

Patients in a range of health care sectors can access their medical health records using a patient portal. In mental health care, the use of patient portals among mental health care professionals remains low. Mental health care professionals are concerned that patient access to electronic health records (EHRs) will negatively affect the patient’s well-being and privacy as well as the professional’s own workload.

**Objective:**

This study aims to provide insights into the appraisal work of mental health care professionals to assess and understand patient access to their EHRs through a patient portal.

**Methods:**

We conducted a qualitative study that included 10 semistructured interviews (n=11) and a focus group (n=10). Participants in both the interviews and the focus group were mental health care professionals from different professional backgrounds and staff employees (eg, team leaders and communication advisors). We collected data on their opinions and experiences with the recently implemented patient portal and their attempts to modify work practices.

**Results:**

Our study provides insights into mental health care professionals’ appraisal work to assess and understand patient access to the EHR through a patient portal. A total of four topics emerged from our data analysis: appraising the effect on the patient-professional relationship, appraising the challenge of sharing and registering delicate information, appraising patient vulnerability, and redefining consultation routines and registration practices.

**Conclusions:**

Mental health care professionals struggle with the effects of web-based patient access and are searching for the best ways to modify their registration and consultation practices. Our participants seem to appraise the effects of web-based patient access individually. Our study signals the lack of systematization and communal appraisal. It also suggests various solutions to the challenges faced by mental health care professionals. To optimize the effects of web-based patient access to EHRs, mental health care professionals need to be involved in the process of developing, implementing, and embedding patient portals.

## Introduction

### Background

The number of patient portals is increasing rapidly in all health care sectors. Through these patient portals, patients have gained the ability to access their medical health records on the internet. A patient portal is a form of eHealth that can be defined as “provider-tethered applications that allow patients to access, but not to control, certain health care information (eg, their EHR [electronic health record]) and provide communication and administrative functions (eg, secure messaging, appointment booking, and prescription refill requests)” [1]. Research has shown that, in mental health care, the use of a patient portal can have a positive effect on patient activation, recovery, and organizational efficiency [2]. In the same study, mental health care professionals were involved during implementation and were trained to use the patient portal [2]. Furthermore, the relationship between the patient and their mental health care professional can improve, provided the mental health care professional has an open attitude, and the medical record is unique, individualized, and detailed [3]. Another study showed that mental health care professionals could feel uncomfortable because they experience reduced control over the information flow when patients can access their health information on the internet [4]. Overall consequences can be positive, for example, improved registration (ie, *documentation*) and consultations (ie, *visits*) with patients or negative, for example, reduced documentation by mental health care professionals. This suggests that the positive effects of web-based patient access partly depend on the registration practices of the mental health care professional and the ways in which they communicate with their patients [3,4]. Therefore, this study explores the appraisal work carried out by mental health care professionals shortly after the introduction of web-based patient access and sheds light on the challenges mental health care professionals face when trying to make a patient portal work for them and the patient. To gain insight into the challenges of mental health care professionals, we use the normalization process theory (NPT), which helps to understand how new technologies and practices are embedded and integrated into existing work practices [5]. This theory “identifies, *characterises* and explains mechanisms that have been empirically demonstrated to motivate and shape implementation processes and affect their outcomes” [6]. NPT includes a model that explains what health care professionals go through when embedding a new technology which, in this study, we have applied to web-based patient access through a patient portal [5-8]. This paper focuses on one of the key constructs of NPT, reflexive monitoring. Reflexive monitoring concerns the appraisal activities that health care professionals do to assess and understand the ways in which a new set of practices affects them and others around them. For patient portals, the focus is on how patients’ web-based access to sensitive data in the EHR affects mental health care professionals, their patients, and the relationship between them. Reflexive monitoring sheds light on the individual mental health care professionals’ appraisal work shortly after the implementation of web-based patient access. Reflexive monitoring involves four components: (1) *systematization*, which involves collecting information about formal (eg, research results) or informal (eg, anecdotal examples) evidence; (2) during *communal appraisal*, individuals work together to evaluate the worth of, in this instance, patient portals and related working routines; (3) through *individual appraisal*, individuals work experientially to appraise the effects on them and the contexts in which they are set; and (4) *reconfiguration* involves attempts to redefine procedures or modify practices, and perhaps, here, even to change the shape of the patient portal itself, to make the patient portal work [5].

Little is known about the appraisal work of mental health care professionals during the embedding of a patient portal. We do know that patient access to medical health records in mental health care has always been a sensitive subject. In the early 1990s, researchers raised the question of whether reading psychiatric case–related notes could be considered offensive [9]. Especially in mental health care, doctors’ notes often contain sensitive information concerning the mental state of the patient [10]. Research suggests that mental health care professionals think there is a risk that patients disagree with the content of the notes or misinterpret the content, and therefore, patients could be upset [9]. This can cause a patient to become concerned or confused and even to respond angrily. In addition to these specific concerns over sensitive information in mental health care, mental health care professionals share the wider concerns of their colleagues in hospital care [10-12]. In total, 2 studies point to a possible higher work burden caused by increased communication with patients and to a fear of lawsuits or claims for damages [10,13]. However, on the other hand, most mental health care professionals believe that patients will better remember their treatment plans and will be better prepared for appointments [10].

### Objectives

This study focuses on appraisal work by mental health care professionals shortly after the implementation of web-based patient access through a patient portal and shows how mental health care professionals try to make sense of this new technology by appraising the effects of the portal and by attempting to modify registrations and consultation practices. Furthermore, our study answers the question of what mental health care professionals do to assess and understand patient access to the EHR through a patient portal.

## Methods

### Overview

For this qualitative study, 10 interviews with a total of 11 mental health care professionals and, later, a focus group, were conducted in a Dutch mental health care organization. This organization (2100 full-time equivalents) offers mental health care, well-being, and social services for approximately 32,000 inpatients and outpatients of all ages. In January 2019, the organization implemented a patient portal for patients to access their EHRs. All patients were able to read notes, letters, and other information in their EHRs after a period of 30 days. Mental health care professionals cannot determine whether a patient uses web-based access. Medical notes were not accessible by patients if they were marked as a draft, but drafts would eventually have to be marked as final before a course of treatment could be closed. After implementation, a personal notes tab was added for the mental health care professionals. These notes were not visible to colleagues or patients.

### Recruitment and Selection

The objective of recruiting study participants was to include mental health care professionals working in diverse focus areas and with different professions within the same mental health care organization. Recruitment, selection, interviews, and focus group were conducted in the spring of 2019. Participants were selected in two ways: by an open invitation on the intranet (n=6) and then through snowballing (n=5). The latter involved asking existing participants if they knew of others who might be willing to be interviewed [14]. All mental health care professionals who expressed willingness to be interviewed were included in the study (Table 1). During the interviews, it became apparent that both supporters and opponents of the patient portal participated in the study.

Participants in the focus group were identified by the head of the computerization and automation department using purposeful sampling (Table 2). This provided a broader range of professions than the interviewee group and included some who had been involved in the implementation of the patient portal.

**Table 1:** Characteristics of the participants of the interviews.

Participant	Sex	Age (years)	Profession	Focus area
1.1	Female	43	Clinical psychologist	Development disorders
1.2	Female	38	Nurse practitioner	Hospital psychiatry
1.3	Male	51	Nurse practitioner	Anxiety and mood
1.4	Male	54	Nurse practitioner	Elderly
1.5	Female	49	Psychiatrist	Personality disorders
1.6	Female	53	Psychiatrist	Addiction
1.7	Female	54	Psychiatrist	Personality disorders
1.8	Female	60	Psychiatrist	Elderly
1.9	Female	27	Psychologist	Development disorders
1.10	Male	39	Psychologist	First level health care
1.11	Female	61	Psychotherapist and team leader care	Forensic

**Table 2:** Characteristics of the participants of the focus group.

Participant	Sex	Age (years)	Profession
2.1	Female	53	Functional application manager
2.2	Female	28	Coordinator health care innovation
2.3	Male	57	Team leader anxiety and mood
2.4	Female	52	Team leader specialist diagnosis and treatment
2.5	Male	50	Functional application manager
2.6	Female	38	Team leader anxiety and mood
2.7	Male	Unknown	Computerization and automation
2.8	Male	37	Client council
2.9^a^	Female	27	Psychologist—development disorders
2.10	Female	37	Strategic marketing and communication advisor

^a^Also an interviewee (participant 1.9).

### Interviews and Focus Group

Before the interviews and the focus group, participants signed an informed consent form and consented to being audio recorded and the use of the data for research.

One researcher (AMvR) conducted the interviews and the focus group, following a predefined topic list (Multimedia Appendix 1), which was based on earlier research on patient portals [11,15,16]. The topic list for the focus group was also based on the results of the interviews’ analysis (Multimedia Appendix 2). During the interviews, participants were asked for their views on and experiences with the potential benefits and risks of patient access to the EHR through a patient portal and possible solutions to reduce the identified risks. The interviews lasted 50 minutes on average (range 30-73 minutes). Most interviews took place in a face-to-face setting. Only one interview was conducted on the internet through Skype because of the geographically distant location of the participant [17].

The focus group was intended to check and enrich the results of the interviews while creating room for elaboration [18]. Participants in the focus group were presented a tentative analysis of the interviews, after which discussion took place according to the predefined topic list (Multimedia Appendix 2). This led to in-depth discussions on the perspectives of mental health care professionals on patient access to their EHR [19]. The focus group lasted 75 minutes and was conducted in a face-to-face setting.

### Analysis

The interviews and the focus group were audio recorded and then transcribed verbatim. First, we followed an inductive approach to analyze the data, in which we repeatedly examined which themes emerged from our data [20]. Second, we took a deductive approach, in which we looked at our data through the lens of NPT to analyze the different components of reflexive monitoring by mental health care professionals. Combined, our analysis can be described as abductive [21]. We coded the data in three steps: open, axial, and selective [22]. Keywords were coupled to certain fragments of the transcripts (Multimedia Appendix 3). Using these keywords, connections were made between different fragments of various transcripts. Thereafter, these keywords were regrouped and formed the basis for drawing conclusions from this research. All the interviews were first individually and separately coded by 2 members of the research team (AMvR and BP), after which these codes and themes were discussed, reviewed, and adjusted if necessary until a consensus was reached (AMvR and BP). Subsequently, we discussed and adjusted the outcomes where necessary with the other members of the research team [20]. The analysis was computer-assisted using ATLAS.ti software (version 8; Scientific Software Development GmbH) [23].

## Results

### Overview

The aim of our study was to provide insights into the appraisal work that mental health care professionals do to assess and understand patient access to the EHR through a patient portal. A total of four interrelated topics emerged from the data analysis: (1) appraising the effect on the patient-professional relationship, (2) appraising the challenge of sharing and registering sensitive information, (3) appraising patient vulnerability, and (4) redefining consultation routines and registration practices.

Our analysis showed that there were both opponents and supporters of web-based patient access among our participants. The following two quotes illustrate the strong differences in opinions among the interviewed mental health care professionals:

I must honestly say that I have not thought about the possible benefits. I only saw disadvantages, felt that I have to be very careful. That was my first response.

P 1.8, psychiatrist

I think it is a greater risk if patients do not have online access.

P 1.5, psychiatrist

Furthermore, our analysis showed that opponents tend to focus on their concerns and have difficulty mentioning the benefits of web-based patient access. When mentioning an advantage, they sometimes immediately denounce the advantages. For example, when asked about the benefit of web-based patient access, one opponent answered:

I might forget to write something down, patients can mention this. So that could be an advantage. However, I must say now that I mention it, I am also immediately afraid that this will cause a lot of extra work.

P 1.8, psychiatrist

### Appraising the Effect on the Patient-Professional Relationship

One of the effects our participants perceived with patient access to the EHR through a patient portal is that it changes the patient-professional relationship.

The first way in which the patient-professional relationship could be changed by patient access is through feedback provided by patients on the content of the EHR. Participants explained that when patients believe the information they read is incorrect or that information is missing, this can be adjusted, leading to therapeutic gain and a new kind of conversation between the patient and professional. One participant illustrated this as follows:

If it [patient access to the EHR] produces complaints, you have to do something about it. If people are correct, they are right to complain and you should not be uncooperative but adjust something. And, it is possible that if you can talk about it with a patient, this could improve the therapeutic relationship.

P 1.4, nurse practitioner

However, participants also mentioned that these extra questions, comments, or even complaints from patients, take time to answer, and mental health care professionals might need to change their records afterwards.

I am a little bit afraid that the people that will be looking [in their medical record], are the people that will have a lot of criticism on what I have written. They will say I did not mean this, I meant it like this.

P 1.8, psychiatrist

Second, our participants argued that patients being able to read their EHR both before and after a consultation with the mental health care professional could enable them to be better prepared for their appointments, and therefore enhance the quality of the conversation between patients and professionals.

Third, participants argued that patients who read the information in their EHR could be more aware of their treatment and feel more like an equal to the mental health care professional. Mental health care professionals could also help create a sense of shared responsibility for the treatment by encouraging patients to study their health information in the patient portal. One participant illustrated the following:

Very often I hear: “Oh, I do not know where my treatment plan is.” You can point it out and mention that it is something that belongs to both of us.

P 1.7, psychiatrist

As the examples above illustrate, our participants believed that web-based patient access could: (1) increase the therapeutic gain, (2) improve the patient’s preparation for a consultation, and (3) improve the involvement of patients in their treatment. However, participants also feared that web-based patient access might cost a lot of valuable time and that patients’ reading notes could have a negative effect on the patient-professional relationship, which is further described in the next section.

### Appraising the Challenge of Sharing and Registering Sensitive Information

Participants admitted that they were struggling with the way they formulated information for the EHR. Medical information in mental health care is often subjective, and writing down a diagnosis is a delicate balance, which is illustrated by one participant’s reflection:

Especially if one [health professional] did not consider it [web-based patient access], one could have written in a somewhat unsophisticated way in the medical record: “This is typical of borderline behavior.,” while not seeing

the patient as borderline.

P 1.7, psychiatrist

Some participants were worried that patients might feel insulted, misinterpret the information given, or feel unheard when reading the information in the EHR, which could reduce trust in the treatment or even withdrawal from the care program:

[...] people who are attached in an unsafe way will very quickly feel let down, and that is also possible through text, which, getting back to the therapeutic relationship, can of course deteriorate, and that would be a pity.

P 1.7, psychiatrist

On the other hand, participants mentioned that such information is an important part of the psychiatric examination and might be important for colleagues to know. If some information is not appropriate for patients to read in their EHR, then mental health care professionals can be reluctant to write it down. One participant illustrated the following:

Let’s assume I see someone who looks dirty or with poor hygiene, then I have a hard time writing that down.

P 1.7, psychiatrist

Besides being subjective, information on mental health care is also often sensitive. Participants argued that patients might become overwhelmed and eventually relapse (a deterioration in the mental health of an individual who was controlling their mental illness) because of the amount or content of the information they have at their disposal with access to their EHR. One participant said:

[...] there are people who can go backwards over small details, such as “I did not study for seven months but eight” [...]

P 1.1, clinical psychologist

This view was confirmed by the participants in the focus group, where a team leader mentioned that he observed that his colleagues were less detailed in their registration:

[...] you also hear that care providers are more aware of what they write in their report, and therefore are more factual and less informative [...]

P 2.3, team leader anxiety and mood

In addition, our participants explained that an mental health care professionals’ report of a consultation might reveal that the patient and the professional had experienced their conversation quite differently and felt differently about what was most important or would therefore summarize the highlights and conclusions differently. Our participants stated that this is not unusual with mental health care and occurs less with physical issues. One participant explained the following:

I wrote a note in the medical record in a certain way, but maybe the other person (the patient) experienced a different conversation.

P 1.7, psychiatrist

Our participants had various views on entering information that is not yet intended for patients. For instance, collateral history might contain sensitive and possibly offensive information and may not always be suitable for patients or known by them. Our participants experience this ethical dilemma: they are not sure whether they should write down sensitive information and whether this information belongs to the patient’s EHR. One participant saw it as a moral dilemma whether to enter certain information or not because it could be beneficial to the treatment but also involves the risk of harming the patient. According to our participants, some information would not be beneficial for patients if they saw it. One participant offered the following example:

I have had a patient, [...] that girl was sixteen years old and her mother was pregnant through the daughter’s boyfriend, and that was written in her medical record, [...] but the girl did not know. [...] It was relevant to the background about the girl’s tangled family situation where all kinds of things had occurred, with very unusual relationships.

P 1.4, nurse practitioner

Another example of doubts about entering information that is not yet, if ever, intended for patients is over certain treatment plans, with participants worrying that they might no longer work if patients can read about them. One participant illustrated a situation where a patient’s husband and her general practitioner thought her situation was deteriorating, but the patient herself did not agree and did not want any kind of treatment. The participant called the patient, and the patient made clear that she did not want any treatment. The participant said the following:

I will make a note of that: “spoken today, clearly different than yesterday, much angrier today, does not want an appointment, does extensively talk about it, agreed that I will call her again next week to see if there are any possibilities then, otherwise I will ask her husband to come here with her,” that is my plan. I did not tell her all of it [...]

P 1.3, nurse practitioner

The issue over treatment plans led to mental health care professionals doubting whether patients should have real-time access to their EHR rather than a 30-day delay. When patients need acute care or are compelled to receive care, for example, in crisis situations, real-time patient access might lead to dangerous situations if patients read what mental health care professionals are planning. One participant stated as follows:

It is possible that when he [the patient] reads this and thinks: “Hey, they are on my doorstep tomorrow [for an involuntary admission], you know what, I will end it [his life] before they arrive.”

P 1.9, psychologist

Despite the dangers of disclosing information to patients, our participants were aware that not entering their thoughts in the EHR also carried risks. Information might otherwise be lost or colleagues are no longer fully informed about certain patients. In crisis situations, where mental health care professionals work in shifts, the peer transfer of information is seen as important by our participants. Furthermore, a participant in the focus group mentioned that mental health care professionals are responsible for what they enter, but also if they fail to enter information that might be of importance later:

[...] suppose you have seen or recognized something, and you did not want to write it down for whatever reason, but it does have an influence on a future course of the treatment, or possibly a crisis situation, and you say: “well, I did see or spot that earlier on,” you are responsible for that.

P 2.4, team leader specialist diagnosis and treatment

This influences the way our participants work individually and together, especially when they disagree about certain issues and have yet to make decisions about how they redefine their registration and consultation practices.

In summary, when sharing and registering delicate information, our participants struggle individually with the way they should write information in the EHR and are afraid that (1) it could reduce patients’ trust in their treatment because patients misinterpret the information they have access to, (2) mental health care professionals might enter information that is inappropriate for patients to read, (3) patients might become overwhelmed by the amount or content of the entered information, (4) it might show to patients that professionals have experienced their conversation quite differently than they did themselves, and (5) there is no place to write down information that is not yet, if ever, intended for the patient to read. This shows that our participants, as individuals, have thought deeply about how to make mental health care patients’ access to their EHR work. There were disagreements over entering information that was not yet, if ever, intended for patients. Whether or not to enter certain information seemed to be a moral dilemma because it could be beneficial to the treatment but also involves a risk of harm to the patient.

### Appraising Patient Vulnerability

Our participants worried that patients could become more vulnerable with web-based access to their EHRs. They were concerned that they had little control over how patients would act on this information in the EHR and are also afraid that patients might, for example, deteriorate after reading their own medical record.

As mentioned in the previous paragraph, information on mental health care is often sensitive. Participants were afraid that this could overwhelm patients and possibly cause a harmful relapse:

[...] during meetings we discuss whether an admission to the ward would be an option. If you write down that you consider this, he [the patient] might get upset or deteriorate. [...] The same goes for our considerations, should we write down something else to prevent a patient from deteriorating?

P 1.9, psychologist

When asked what is meant by deterioration, a participant answered as follows:

[...] a patient getting completely disordered, mentally stuck, upset, a breach of trust with their mental health care professionals [...].

P 1.1, clinical psychologist

Participants explained that patients could easily print or download their own medical records, after which they could share this with *inappropriate* people. In this way, sensitive information may fall into the wrong hands. A third party, such as a curious spouse, could also gain access to the EHR for wrong reasons. Especially in mental health care, patients are often vulnerable and easily influenced by relatives. One participant stated as follows:

A disadvantage could be that someone else gets access to the password or login codes, that could of course be a risk. With certain treatments, you do not want a partner to know certain things, [...] however they [relatives] can be persuasive and demand access from a patient.

P 1.10, psychologist

Our participants were unsure who would be responsible for the potentially reckless handling of information from the EHR by the patient. They also doubted whether it would be sufficient if mental health care professionals warn patients about the sensitive nature of the information. One participant, however, stated that sharing health data was the responsibility of the patients:

The patient has access, so I think it is their responsibility. I think the content and the correctness of the content is the responsibility of the health professional.

P 1.10, psychologist

Another participant mentioned an extreme example of what could happen when patients share their own medical information, for example, to show that they are discontent with their treatment, but emphasized that this is the patient’s own responsibility:

If the patient thinks: “I will go to Story or RTL boulevard [national media] with my medical record, which sometimes happens, then they can do it.”

P 1.11, psychotherapist and team leader care

In summary, our participants were afraid that they had much less control over what patients do with the information in the EHR and wonder who is responsible for sharing information. It seems that patient access raises many uncertainties concerning individual personal relationships with a patient.

### Redefining Consultation Routines and Registration Practices

Reflecting on the effects and struggles of entering sensitive information in an EHR, our participants suggested various solutions in terms of modifying their registration practices. However, those who opposed the idea of web-based patient access were not convinced that those solutions would really work, as they often also mentioned the possible disadvantages of the suggested solution.

#### Solution 1: Draft Notes for Colleagues

The first solution suggested by the participants was to write draft notes for colleagues. This is a temporary solution, in that draft notes will not be immediately visible in the patient portal but will need to be marked as final, and hence become visible, before a treatment can be closed.

#### Solution 2: Making Personal Notes Visible for Colleagues

The second solution was to make the personal notes tab visible to colleagues. Although this prevents the loss of access to information in, for example, crisis situations, this also reduces the transparency of information for patients because a hidden *shadow file* is created.

#### Solution 3: Discussing Information With Patients Before Registration

A third suggested solution was to discuss information with patients before the mental health care professionals register this information. In this way, they can ensure that there is no new information in the EHR should the patient choose to access it. A participant in the focus group explained this as follows:

[...] it is quite difficult in that you cannot write down your considerations, but I think it is also a stimulant to share your considerations with the patient a lot more, by which you give a patient more space and influence, which causes the treatment relationship to become more equal [...].

P 2.6, team leader anxiety and mood

However, participants acknowledged that this third option was only workable if they discuss the information directly during a consultation. If not, if mental health care professionals delay registration, this increases the risk of mistakes and lost information because of memory shortfalls. At the same time, our participants commented that they often let a conversation sink in and write the report later:

[...] of course it remains difficult, when you walk back into your room and you smell alcohol [lingering from the patient] after the end of a consultation. Where do you record this, as you have not yet discussed it with the patient, but it is important information, these are difficult things.

P 2.6, team leader anxiety and mood

#### Solution 4: Registering Information Together With the Patient

A fourth solution that is mostly mentioned by supporters is the practice of registering information together with the patient. This collaborative practice could even become a form of treatment. Our participants felt that it depends on the patient whether this would be a workable solution, and two possible obstacles were raised by opponents. First, it was noted by the participants that certain patients (eg, psychotic patients or those with developmental disorders) are not capable of writing notes along with their mental health care professional. For example, an opponent mentioned that patients with a developmental disorder are often overstimulated after a consultation and would not be able to contribute to writing notes:

The argument is: “you have to write [in the medical record] together with your patient, use the last ten minutes of your consultation.” However, that does not work with our patients. They are completely overstimulated after half an hour, they cannot immediately reflect on what happened.

P 1.1, clinical psychologist

However, when a supporter was confronted with this concern, she responded as follows:

It can be an extra effort, but that is also part of the dynamics of that treatment. [...] No psychiatrist is made to treat everyone, [...], so I guess choose your patient population according to that.

P 1.5, psychiatrist

Second, some participants were concerned that writing notes with the patient would eat into the already limited time for consultation.

#### Solution 5: Introducing Patients to Web-Based Access at the Beginning of Treatment

The final solution was to introduce patients to web-based access to their EHRs at the beginning of their treatment. Mental health care professionals could then explain the risks and benefits of web-based patient access and decide together with the patient whether the patient would use it. One participant said the following:

Sometimes the risks have to be pointed out to a patient, as I just said, you can send a copy of your letter but watch out when you use it in court, so they need to be informed about the risks.

P 1.6, psychiatrist

Our participants would like more support on what kind of information patients can read in the EHR and how they should write sensitive information in the EHR. This could provide them with more knowledge and enable them to experiment with web-based patient access and to evaluate the outcomes together. Our participants said it was unclear to them what kind of information patients could read through the patient portal and on which terms. Furthermore, participants commented that they had only limited experience with the patient portal because it had only just been implemented. Indeed, most participants had no personal experience with patients accessing their EHR at all. However, some participants were able to report on one encounter with a patient who had read their EHR and then regretted doing so:

Some patients get overwhelmed by the amount of information, one patient said the following: “I just regret looking because I started and I got so much information, well, I got really upset, then I stopped.”

P 1.7, psychiatrist

As illustrated earlier, our participants were individually able to come up with five solutions that they believed could make patient access to the EHR work for them as well as for their patients. However, it would appear that our participants needed more support on how the portal works so that they could actually experiment with their ideas on working with web-based patient access and evaluate these experiments.

## Discussion

### Principal Findings

This study seeks to provide insights into the appraisal work that mental health care professionals do to assess and understand patient access to their EHRs through a patient portal. By interviewing 11 mental health care professionals and conducting a focus group discussion, we learned that mental health care professionals struggle with how to weigh up the potential benefits and risks they perceive and are trying to work out what they can do themselves to make the portal work for them and for their relationship with their patients.

Our results show that mental health care professionals struggle with various aspects of patient access to the EHR and with entering what they perceive as sensitive information into the EHR. First, we looked at the ways in which mental health care professionals appraise the effect of web-based patient access on their relationship with the patient. Second, we report how mental health care professionals fear that some patients are too vulnerable to handle the new possibility of accessing their medical records. Third, we showed the ways in which mental health care professionals address the challenge of registering and discussing delicate information. Finally, we showed how mental health care professionals individually experiment by redefining consultation routines and registration practices.

Our results show that participants are actively engaged in the NPT terms reflexive monitoring, especially the components related to individual appraisal and reconfiguration [5]. Our participants individually appraised the effects of patient access to the EHR (eg, that mental health care professionals should perhaps no longer write so freely in the medical record) and thought about solutions to modify and redefine their registration and consultation practices. Participants mentioned that notes might become less accurate and less detailed to avoid potential harm to the patient, a concern also expressed elsewhere in the literature [10,13,24]. Although some studies show that, in practice, only very few patients are actually harmed [12,25], another study showed that patients could be surprised or hurt when they read information in the medical record that is incorrect, outdated, or new to them [3]. Such patients are then afraid that this incorrect or outdated information might have a negative impact on their treatment if, for example, other mental health care professionals read and act on this information [3]. Other patients commented that this makes them doubt whether their mental health care treatment is useful [3]. The other two NPT components, systematization and communal appraisal, did not appear to take place. As long as mental health care professionals struggle to engage with these two components of reflexive monitoring, embedding web-based patient access in the work practices of mental health care professionals will be hindered. Consequently, we hope that future research will explore the ways in which systematization and communal appraisal can be stimulated during the implementation of web-based patient access in mental health care. In addition, future research could focus on ways to involve opponents of web-based patient access in the process of communal appraisal and reconfiguration.

Furthermore, our results show that participants worry that certain treatment plans and strategies might no longer work if patients can read them. This is a new concern that has not been mentioned in the literature before and is especially relevant as information in the EHR becomes accessible in real time. However, a study on real-time access through a patient portal in hospital care concluded that the limited negative consequences could be mitigated by instruction, education, and preparation of patients by the mental health care professionals [26]. Further research on this topic in mental health care is recommended and could focus on the cocreation of further development of web-based patient access with patients [27].

NPT suggests that appraisal work needs to include communal appraisal if a technology is to become normalized, that is, for it to become an integrated aspect of the mental health care professionals’ work routines. During the interviews, participants suggested various solutions to the struggles they experience with patients having web-based access to their medical health records. Individual mental health care professionals suggesting adaptions to the new service, so that it becomes a normalized practice, is in accordance with the reflexive monitoring component of NPT [5]. Mental health care professionals and the organization as a whole could work on these solutions to eventually embed web-based patient access in their daily work routines. For this to occur, mental health care professionals can discuss their concerns and struggles and cocreate solutions, such as the concerns and solutions expressed and suggested during the interviews [28]. The solutions mentioned in this paper could serve as a starting point but still need to be evaluated in practice.

### Limitations

Our study has four limitations. First, our study focused on a specific organization in mental health care with the mental health care professionals involved all having a similar amount of experience with web-based patient access. Furthermore, not all focus areas within mental health care were represented. Therefore, some mental health care divisions, such as forensic psychiatry and primary mental health care, were probably underrepresented. Second, because all the participants actively responded to an open invitation to participate, there is a risk of selection bias. There is also a possibility that only early adopters of the patient portal participated in the interviews, given that the organization implemented the portal in January 2019, and the interviews were conducted in the spring of that year. It might be possible that the participants of this study were not representative of the population of mental health care professionals. However, the interview transcripts show that both proponents and opponents and some mental health care professionals with more neutral views took part in the study. Moreover, it is important to note that, given the very limited time between the implementation of the patient portal and our interviews, most of the worries expressed by the participants were not based on specific personal experiences with web-based patient access. It would be interesting to repeat this study to see whether the mental health care professionals have changed their minds or have experienced the struggles they expected and whether collective experience or evaluations had already occurred. A third limitation is that, apart from one participant in the focus group, the patient perspective was excluded. Further research is needed to explore how the doubts expressed in this study are experienced by patients. Finally, in the topic list, we choose not to explicitly ask participants to reflect on the four different components of reflexive monitoring according to NPT. In contrast, we chose to center the appraisal activities as articulated by the participants themselves. Future research is needed to validate our finding that systematization and communal appraisal are not the predominant components of reflexive monitoring by mental health care professionals.

### Comparison With Prior Work

Research shows that patient access through patient portal empowers patients, meaning that patients feel more in control of their mental health care [2,12,29]. A pilot study involving 52 psychiatric patients gaining web-based access to their medical health record found that 82% of the included patients felt more in control of their own treatment because of the possibility of reading their treatment plans and medical notes and knowing what they could expect in their care process [12]. However, our results show that doubts remain as to whether mental health care patients can handle access to their own EHR. For example, our participants were afraid that patients might share their medical records with an unauthorized person or authority, which could make patients more vulnerable to people or institutions with conflicting interests. A recent review similarly raised this concern regarding patients autonomously handling medical information [30]. Another study found that a major barrier to redefining work practices of health care professionals through the use of patient portals in hospital care concerned privacy and security [31]. These examples support our finding that mental health care professionals are struggling to assess and understand the effect of web-based patient access for their patients and their work practices. Further research should confirm our findings and should look for more solutions to reduce the privacy and security concerns of mental health care professionals.

There is a moral dilemma if the benefits of web-based patient access are associated with an increase in patient vulnerability. This has its roots in the normative question of what is *good*. Is it *good* to aim for the benefits of web-based access and increasing empowerment, but possibly also resulting in an increase in patient vulnerability, or is it *good* to prevent an increase in vulnerability that involves withholding possible benefits? And, maybe even more importantly, whose decision is this to make? There are no universal answers to these normative questions, but it is important to recognize and discuss these dilemmas. The thin line between patient autonomy, patient empowerment, and patient vulnerability has been discussed in various studies on patient-centered care, as is evident from a discourse analysis on patient-centeredness, which indeed highlights that there are different views on what is *good* patient care [32]. Some consider patient-centeredness to be a process of *empowering patients*, implying that they believe patients should be given the possibility to view their medical data on the web. Withholding web-based access to medical information for vulnerable patients could be considered unethical in this discourse. Risks are recognized, but empowerment also helps patients to appropriately deal with the risks of web-based patient access. In another discourse, which we label *caring for patients*, people have a more paternalistic view of patient-centeredness and believe that health care professionals should protect patients from risks. Our results indicate that some mental health care professionals doubt that it is their task to protect patients from certain vulnerabilities. However, our participants also commented that not all patients are the same and that patients require tailored care. This reflects the *being responsive* discourse, which argues that patient-centered care is about meeting the specific and highly differing needs of patients. Individual mental health care professionals and organizations as a whole need to determine what patient-centered care means to them and how they want to deal with the moral dilemmas associated with patient access to the EHR. Communal appraisal can be arranged by organizing a moral deliberation, one of the ways to organize a dialog about the moral dilemmas of patient autonomy versus patient vulnerability [33].

As our results indicate, the protection of vulnerable patients might not only be the responsibility of mental health care professionals through individual appraisals. The literature shows that this can also be achieved through laws and regulations [34]. Patients gaining more control over their own EHR falls under the term *informational self-determination*, which is defined as “the ability of a person to determine, in principle, to what extent personal data is used and further disclosed, in view of a self-determined life” [34]. With an increase in informational self-determination, the risk of spreading medical information to parties who are not entitled to it increases. A possible solution could be to implement patient confidentiality, in which medical information managed by patients is legally protected [34]. Further research could explore the feasibility of this concept and look at ways to include mental health care professionals and modify their practices.

Research investigating patient access through a patient portal in hospital care has shown that patients’ interests and abilities in using a patient portal are influenced by various factors, including age, health literacy, and level of education [35]. Patients are more likely to use a patient portal if it suits their information needs and has the functionalities they require [35]. Our results show that a possible solution could be to introduce every new patient to web-based patient access with mental health care professionals, discussing with them the possibilities and the possible risks regarding privacy and their responsibilities. This would involve mental health care professionals in (1) collecting information in various ways, such as asking the opinions of patients and colleagues; (2) jointly evaluating how introducing new patients to web-based patient access would work; (3) individually experiencing if introducing every new patient to web-based patient access adds value; and (4) appraising, alone or with each other, if this way of working requires a redefinition of their registration and consultation practices, or even a change in the patient portal itself. In a recent study, some mental health care professionals believed that informing patients about the benefits and risks of reading medical notes was worthwhile [36]. However, in the same study, there were also mental health care professionals who were reluctant to inform patients about this because they feared negative outcomes. The study concluded that clear patient-professional communication about web-based access to medical information would prevent potential harm. However, another study concluded that introducing every patient to web-based access at the beginning of their treatment would be time consuming and might not be feasible [10]. Another option would be a web-based educational program for mental health patients to introduce them to web-based access. Indeed, one study argued that this may help empower patients and increase their active participation in their own care [37]. Another study found that a web-based course for mental health care professionals on web-based patient access in mental health care resulted in a reduction in mental health care professionals’ worries about web-based patient access and an improvement in aspects of patient-professional communication [37]. Further research is needed to explore the feasibility of these solutions as a way to modify the practices of mental health care professionals; researchers should also be open to other possible solutions, such as action research, because this can directly improve the embedding of patient EHR access because improvements can be made during the study [38].

### Conclusions

This study provides insights into the appraisal work that mental health care professionals do to assess and understand patient access to their EHRs through a patient portal. Our study explores and describes the effects and struggles that mental health care professionals experience with patients having access to their EHR and how they individually experiment to redefine and modify their work practices. One new insight, not previously reported, is that mental health care professionals are concerned that their treatment plans might no longer be effective. In certain situations, such as when patients need acute care or are compelled to receive care, real-time patient access might lead to dangerous situations because patients act before mental health care professionals can carry out their treatment plan. Furthermore, our study signals a lack of systematization and communal appraisal. Our participants predominantly seem to individually appraise the effects of web-based patient access and how they can modify their registration and consultation practices. Future research is needed to investigate the ways in which systematization and communal appraisal can be stimulated.

In addition, future research could investigate the viability of the modifications in consultation routines and registration practices proposed by our participants. Finally, future research could focus on ways to involve opponents of web-based patient access in communal appraisal. The findings of this study can help researchers, project leaders, project staff, policy officers, and mental health care professionals to understand the process of embedding a new technology and the need for communal appraisal. To further improve working with web-based patient access, mental health care professionals need to be involved in evaluations and the further development of patient portals.

## Appendix

Multimedia Appendix 1 Topic list for the interviews.

Multimedia Appendix 2 Topic list for the focus group.

Multimedia Appendix 3 Analysis of keywords.

## Abbreviations

EHR: electronic health record
NPT: normalization process theory
