# Supplementary material for: Mental Health Care Professionals’ Appraisal of Patients’ Use of Web-Based Access to Their Electronic Health Record: Qualitative Study
Source: J Med Internet Res. 2021 Aug 27;23(8):e28045. doi: 10.2196/28045 (PMC8433850; doi:10.2196/28045)
Supplement: Multimedia Appendix 1 [file jmir_v23i8e28045_app1.docx]

# Topic list interviews

**Purpose**

**Risks of online access through a patient portal
Solutions to reduce risks of online access**

**Introduction**

- Erasmus University Rotterdam
- Explanation of the interview (duration, transcript)
- Recording and privacy
- Drop-off
- Introduction on online access through a patient portal

**Personal introduction**

**Patient portals in general**

- Definition of a patient portal
- Actual functions of a patient portal
- Required functions according to participants
- Initial reaction on the implementation of online access
- Experience with online access

**Performance expectation**

- Added value of online access within mental healthcare (therapy adherence, clinical outcomes, communication, patient satisfaction)
- Impact of online access on activities (quality, productivity, efficiency)
- Time-saving due to online access
- Change in responsibility
- Use of online access within treatment, recommending use with patients
- Other concerns on the added value of online access
- Solutions for risks of online access within performance expectation

**Effort expectancy**

- User-friendliness of online access
- Learning curve
- Impact of online access on workload
- Questions on online access
- Other concerns about using online access
- Solutions for risks of online access within effort expectancy

**Social influence**

- Opinion colleagues
- Encouraging use of online access
- Attitude management when using online access
- Autonomy in use of online access
- Other concerns about social influence in online access
- Solutions for concerns about social influence

**Facilitating Conditions**

- Sufficient knowledge to use the patient portal (knowledge of legislation and rules, what should and what should not be written down in patient records, responsibility)
- Resources available to use the patient portal
- Integration with other systems
- Support
- Instructions for use
- Fits work style
- Other concerns about facilitating conditions for online access
- Solutions for concerns about facilitating conditions

**Other concerns with online access**

- Harming patients through online access
- Writing in the medical record as medical professional
- Positive effect of online access
- Negative effect of online access
- Concerns in practice

**Forgotten or underexposed subjects**

**Experience interview**

**Focus group**

**End of interview**
