# Supplementary material for: Mental Health Care Professionals’ Appraisal of Patients’ Use of Web-Based Access to Their Electronic Health Record: Qualitative Study
Source: J Med Internet Res. 2021 Aug 27;23(8):e28045. doi: 10.2196/28045 (PMC8433850; doi:10.2196/28045)
Supplement: Multimedia Appendix 2 [file jmir_v23i8e28045_app2.docx]

# Topic list focus group

**Purpose**

**Risks of online access through a patient portal**

**Solutions to reduce risks of online access**

**Introduction**

- Erasmus University Rotterdam
- Explanation of the focus group (duration, transcript)
- Recording and privacy
- Drop-off
- Introduction on online access through a patient portal

**Personal introduction**

**Patient portals in general**

- Definition of a patient portal
- Actual functions of a patient portal
- Experience with online access

**Risks of online access known in literature**

- Increase in workload
  - Insufficient compensation
- Technical skills
  - Missing important information
- Responsibility
  - Data breach
  - Harmful behavior of patients
- Autonomy

**Solutions for risks known in literature**

- Way of writing in the medical record
  - Transparency towards the patient
  - Descriptive, non-judgmental and summarizing, emphasizing strengths
  - Psychiatric examination
  - Describe non-pathological qualities
- Involve patients in treatment
  - Involvement in online access, discussion about experience
  - Involvement in documenting in the medical record

**Risks of online access experienced by medical professionals**

- Workload
  - Fear of increase in workload
  - Spend more time on documenting in the medical record
- Outcomes
  - Doubts about effect on clinical outcomes
  - Harmful for patients
    - Old medical notes are also accessible
    - Overwhelming for patients
  - Treatment plans longer effective
  - No area for personal notes
    - Loss of information
    - Transfer with colleagues
- Therapeutic relationship
  - Psychiatric examination and documentation, interpretation
  - Explanation of notes when viewing a copy on paper
- Patient empowerment
  - Value of self-management
  - Privacy sensitive information
- Instructions and guidelines
  - Writing medical notes
  - Legal responsibility (data breach, harmful for patient)

**Solutions to risks experienced by medical professionals**

- Workload
- Personal work notes
  - Write information down as soon as it has been discussed
  - Shadow file, concept / final, other terminology “phimosis cerebri”
  - Insightful for colleagues
  - Way of writing notes
- Therapeutic relationship
  - Difference in experience and interpretation of patient / medical professional
  - Writing notes together
    - Difference in patients
    - Settling of information
    - Treatment time
- Patient empowerment
  - Involve in treatment
  - Privacy
    - Information, warning
    - Medical professional indicates what may be viewed by other authorities
- Instructions and guidelines
  - Guideline for writing medical notes from the organization
  - Guideline (legal) responsibility

**Forgotten or underexposed subjects**

**Experience focus group**

**End of focus group**
