# Supplementary material for: Mental Health Care Professionals’ Appraisal of Patients’ Use of Web-Based Access to Their Electronic Health Record: Qualitative Study
Source: J Med Internet Res. 2021 Aug 27;23(8):e28045. doi: 10.2196/28045 (PMC8433850; doi:10.2196/28045)
Supplement: Multimedia Appendix 3 [file jmir_v23i8e28045_app3.docx]

# Keywords analysis

Adoption process: implemented in consultation with mental healthcare professionals

Adoption process: way of registration

Adoption process: focus on technique

Adoption process: information on patient portal: flawed according to mental healthcare professional

Adoption process: getting used to

Definition patient portal: naming

Higher workload: by registering different

Higher workload: by criticism or questions from patients

Impact on patient-professional relationship: miscommunication

Impact on patient-professional relationship: complaints

Access rights: procedure

Access rights: role of caregivers or family members

Access rights: incapacitated patients

Solution: discuss what is being registered

Solution: delicate information marked as concept

Solution: discuss privacy concerns with patient

Solution: instructions from professional association

Solution: discuss access rights care givers

Solution: writing registration together with patient

Solution: stimulating usage among patients

Solution: further developments patient portal

Solution: sharing personal notes

Reaction patient: no need for online access

Reaction patient: still unclear

Reaction patient: unclear wish

Reaction patient: information was unclear

Reaction patient: varying

Challenges for patient: availability computer

Challenges for patient: digital skills

Challenges for patient: risk of anxiety

Challenges for patient: worries about privacy

Challenges for patient: worries about privacy: family issues

Challenges for patient: worries about privacy: parent-child issues

Change in registration: personal versus business style

Change in registration: linguistic

Change in registration: working hypothesis

Benefits: meeting patient information needs

Benefits: consultation preparation by patient

Benefits: no prospect of potential benefits

Benefits: increase in patient empowerment

Benefits: transparency

Benefits: patient contributes to health record

Benefits: patient is aware of agreements

Benefits: patients completes what professional has forgotten

Benefits: time saving: faster communication

Benefits: time saving

Benefits: therapy adherence
